# Sumoylation-deficient phosphoglycerate mutase 2 impairs myogenic differentiation

**DOI:** 10.3389/fcell.2022.1052363

**Published:** 2022-12-14

**Authors:** Yi Zhang, Ilimbek Beketaev, Yanlin Ma, Jun Wang

**Affiliations:** ^1^ Hainan Provincial Key Laboratory for Human Reproductive Medicine and Genetic Research, Reproductive Medical Center, The First Affiliated Hospital of Hainan Medical University, Hainan Medical University, Haikou, China; ^2^ Stem Cell Engineering, Texas Heart Institute, Houston, TX, United States; ^3^ Key Laboratory of Tropical Translational Medicine of Ministry of Education, Hainan Medical University, Haikou, China

**Keywords:** phosphoglycerate mutase 2, sumoylation, posttranslational modification, myogenic differentiation, mitochondrial function

## Abstract

Phosphoglycerate mutase 2 (PGAM2) is a critical glycolytic enzyme that is highly expressed in skeletal muscle. In humans, naturally occurring mutations in Phosphoglycerate mutase 2 have been etiologically linked to glycogen storage disease X (GSDX). Phosphoglycerate mutase 2 activity is regulated by several posttranslational modifications such as ubiquitination and acetylation. Here, we report that Phosphoglycerate mutase 2 activity is regulated by sumoylation—a covalent conjugation involved in a wide spectrum of cellular events. We found that Phosphoglycerate mutase 2 contains two primary SUMO acceptor sites, lysine (K)49 and K176, and that the mutation of either K to arginine (R) abolished Phosphoglycerate mutase 2 sumoylation. Given that K176 is more highly evolutionarily conserved across paralogs and orthologs than K49 is, we used the CRISPR-mediated homologous recombination technique in myogenic C2C12 cells to generate homozygous K176R knock-in cells (PGAM2^K176R/K176R^). Compared with wild-type (WT) C2C12 cells, PGAM2^K176R/K176R^ C2C12 cells exhibited impaired myogenic differentiation, as indicated by decreased differentiation and fusion indexes. Furthermore, the results of glycolytic and mitochondrial stress assays with the XF96 Extracellular Flux analyzer revealed a reduced proton efflux rate (PER), glycolytic PER (glycoPER), extracellular acidification rate (ECAR), and oxygen consumption rate (OCR) in PGAM2^K176R/K176R^ C2C12 cells, both at baseline and in response to stress. Impaired mitochondrial function was also observed in PGAM2^K176R/K176R^ P19 cells, a carcinoma cell line. These findings indicate that the PGAM2-K176R mutation impaired glycolysis and mitochondrial function. Gene ontology term analysis of RNA sequencing data further revealed that several downregulated genes in PGAM2^K176R/K176R^ C2C12 cells were associated with muscle differentiation/development/contraction programs. Finally, PGAM2 with either of two naturally occurring missense mutations linked to GSDX, E89A (conversion of glutamic acid 89 to alanine) or R90W (conversion of arginine 90 to tryptophan), exhibited reduced Phosphoglycerate mutase 2 sumoylation. Thus, sumoylation is an important mechanism that mediates Phosphoglycerate mutase 2 activity and is potentially implicated in Phosphoglycerate mutase 2 mutation-linked disease in humans.

## Introduction

Phosphoglycerate mutases (PGAMs) are a group of critical enzymes that convert 3-phosphoglycerate (3-PG) to 2-phosphoglycerate (2-PG) (Fothergill-Gilmore and Watson, 1989) in the glycolytic pathway. By controlling the amount of 3-PG and 2-PG in cells, PGAMs play an important role in glycolysis and the biosynthesis of molecules essential for normal cellular function and survival. The PGAM family contains three members: PGAM1, the brain isoform (also named PGAM-B); PGAM2, the muscle isoform (also named PGAM-M); and PGAM3 (also named PGAM4), the testes isoform (Okuda et al., 2012). Among these three isoforms, PGAM3 is derived from the retrotransposition of the *PGAM1* gene and is believed to be functional (Betran et al., 2002; Okuda et al., 2012). PGAM2 is the only gene among these three that is etiologically linked to glycogen storage disease X (GSDX) in humans. Two naturally occurring missense mutations of PGAM2 have been identified: the mutation of glutamic acid residue 89 to alanine (E89A) and the mutation of arginine residue 90 to tryptophan (R90W) (Naini et al., 2009). PGAM2 is highly enriched in heart and skeletal muscle. (Shanske et al., 1987). In mice that conditionally express PGAM2 in the heart, no discernible phenotypes were observed while at homeostasis. However, the tricarboxylic acid (TCA) cycle was altered in these mice, (Okuda et al., 2013), and they developed systolic dysfunction in response to pressure overload (Okuda et al., 2013). Although PGAM2 is highly expressed in skeletal muscle, it remains unknown whether and how PGAM2 plays a role in myogenic differentiation, a critical process in muscle development and regeneration after injury.

Studies in cancer cells have shown that PGAM2 plays a significant role in the Warburg effect (Mikawa et al., 2020) (i.e., increased glycolysis, a characteristic of cancer cells). However, PGAM2 is one of few energy metabolism–related enzymes that does not undergo transcriptional regulation by heat-induced factor 1 alpha (HIF1a), which is a master regulator of energy metabolism in cancer cells under hypoxia (Mimeault and Batra, 2013; Nagao et al., 2019). Therefore, posttranslational modifications (PTMs) have been speculated to mediate PGAM2 activity. Indeed, acetylation of lysine 100 (K100) on PGAM2 suppresses its activity, whereas deacetylase sirtuin 2 (Sirt2) deacetylates and stimulates PGAM2 activity (Xu et al., 2014). Moreover, PGAM2 acetylation is mediated by oxidative stress, which potentiates Sirt2 activity and decreases PGAM2 acetylation (Xu et al., 2014). In addition, ubiquitination of PGAM2 mediated by Mdm2 promotes PGAM2 degradation *in vitro* (Mikawa et al., 2014).

Conjugation by small ubiquitin-like modifiers (SUMOs), called SUMO conjugation or sumoylation, is the covalent attachment of SUMO proteins to substrates through enzymatic cascades. The SUMO family has five SUMO isoforms, namely SUMO-1, -2, -3, -4, and -5. At the amino acid level, SUMO-1 exhibits high homology with SUMO-5, and SUMO-2 exhibits high homology with SUMO-3, whereas SUMO-1 shares approximately 45% similarity with SUMO-2/3 (Diao et al., 2009; Liang et al., 2016). Although SUMO-4 is non-conjugable in its natural form (Owerbach et al., 2005), it is believed to play a role in some diseases such as diabetes mellitus (Bohren et al., 2004; Aribi, 2008). SUMO-1 and SUMO-2/3 have common but also distinct targets (Vertegaal et al., 2006) and therefore may play different roles in cellular events and disease pathogenesis. For instance, HDAC1 is targeted by SUMO-1 but not by SUMO-2 in the myogenic C2C12 cell line (Joung et al., 2018).

SUMO conjugation has emerged as a versatile PTM involved in a wide spectrum of cellular events that may contribute to disease pathogenesis. For example, several naturally occurring mutants of Zic3, a transcription factor involved in congenital malformations such as neural tube defects, display deficient SUMO conjugation (Chen et al., 2013). In addition, studies have shown an important role for SUMO conjugation in myogenic differentiation and myotube fusion. In support of this, the knockdown of Ubc9, which is the sole E2 in the sumoylation pathway, severely impairs the terminal differentiation of C2C12 cells without affecting the expression of critical muscle-related factors such as MyoD and myogenin (Riquelme et al., 2006). Furthermore, suppression of Nse2, which is a SUMO E3 ligase, delays myogenic differentiation (Berkholz et al., 2014). Intriguingly, while levels of SUMO-1 and SUMO-2/3 conjugation increase in confluently cultured C2C12 cells, myogenic differentiation reduces global SUMO conjugation (Liu et al., 2020). At the individual SUMO substrate level, HDAC1 sumoylation dissociates HDAC1 from MyoD, thereby releasing its inhibition of MyoD and promoting myogenesis (Joung et al., 2018). Together, these observations support the premise that SUMO conjugation plays an important role in myogenic differentiation.

We report here that PGAM2 is modified by SUMO-1 on two highly conserved lysine residues, K49 and K176. In C2C12 cells with the homozygous knock-in of K176R, myogenic differentiation and mitochondrial function were impaired. Moreover, PGAM2 with naturally occurring missense mutations that are etiologically linked to GSDX in humans resulted in decreased sumoylation compared with wild-type PGAM2. Our findings support that sumoylation is an important PTM that regulates PGAM2 activity.

## Materials and methods

### Antibodies and reagents

Horseradish peroxidase (HRP)-conjugated β-actin antibody (Cat# sc-47724, 1:20000) was from Santa Cruz Biotechnology, Inc. (Dallas, TX, United States). Rabbit anti-PGAM2 antibody (Cat# A7917, 1:1000) was from ABclonal (Manhattan Beach, CA, United States). The rabbit anti-caveolin 3 antibody (Cat# NB110-502, 1:5000) was from Novus Biologicals (Centennial, CO, United States). The anti-sarcomeric α-myosin heavy chain (α-MHC) antibody MF20 (Cat#14-6503-82, 1:500) was obtained from eBioscience, Inc. (San Diego, CA, United States). HRP-conjugated HA antibody (anti-HA-HRP, Cat# A00169, 1:1000) was from Genscript (Piscataway, NJ, United States). The fluorophore-conjugated secondary antibodies Alexa Fluor^®^ 546 (goat anti-mouse, Cat# A-11003, 1:500), lipofectamine 2000, lipofectamine 3000, V5-HRP, and NuPAGE precast gels were from ThermoFisher Scientific (Carlsbad, CA, United States). T4 polynucleotide kinase, QuickLigase kit, DH5α cells, and NEBNext High-Fidelity 2xPCR Master Mix were from New England Biolabs (Ipswich, MA, United States). The QuickChange II XL site-directed mutagenesis kit was purchased from Agilent (La Jolla, CA, United States). ECL Plus was from GE Healthcare (Chicago, IL, United States). M2-HRP, Immobilon™ Western, and polyvinylidene difluoride (PVDF) membrane were from MilliporeSigma (Temecula, CA, United States). Nickel-nitrilotriacetic acid (Ni-NTA) beads and Mini Prep Kits were purchased from Qiagen (Germantown, MD, United States). Dulbecco’s Modified Eagle’s Medium (DMEM) was purchased from Corning, Inc. (Corning, NY, United States). Fetal bovine serum (FBS) was purchased from Atlanta Biologicals (Flowery Branch, GA, United States).

### Plasmid constructs

Expression vectors encoding HA-his_6_-tagged SUMO-1 wild-type (WT) (HA-SUMO-1-wt) and HA-his_6_-tagged SUMO-1-ΔGG mutant (HA-SUMO-1-ΔGG-mut, deficient for conjugation); HA-his_6_-tagged SUMO-2-WT (HA-SUMO-2-wt) and HA-his_6_-tagged SUMO-2-ΔGG mutant (HA-SUMO-2-ΔGG-mut, deficient for conjugation); flag-tagged SUMO-1-WT (flag-SUMO-1-wt) and flag-tagged SUMO-1-ΔGG-mut (flag-SUMO-1-ΔGG-mut); flag-tagged SUMO-2-WT (flag-SUMO-2-wt) and flag-tagged SUMO-2-ΔGG mut (flag-SUMO-2-ΔGG-mut); flag-tagged SENP2-WT and SENP5-WT and enzymatically inactive mutants (flag-SENP2-wt, flag-SENP2-mut, flag-SENP5-wt, flag-SENP5-mut); and HA-tagged PIAS1, 2α, 2β, 3 and 4 were described previously (Wang, 2010; Chen et al., 2013; Beketaev et al., 2014; Kim et al., 2015a). The V5-his_6_-tagged PGAM2 expression vector was generated by performing PCR amplification of mouse PGAM2 cDNA (Clone# 4235174) from Horizon Discovery (St. Louis, MO, United States) by using the following two oligos: forward, 5′ CAC​TAG​TCC​AGT​GTG​GTG​GAA​TTC​atg​acc​acc​cac​cgc​cta​gt 3′, and reverse 5′ TTC​GAA​GGG​CCC​TCT​AGA​CTC​GAG​ctt​cgc​ctt​tcc​ctg​ggc​a3′, followed by subcloning into pcDNA4A-V5-his_6_ vector with EcoRI and XhoI sites (designated as PGAM2-V5-his). Similarly, the HA-tagged PGAM2 expression vector (HA-PGAM2) was generated by performing PCR amplification of mouse PGAM2 cDNA by using the following oligos: forward, 5′AGC​CTG​GGA​GGA​CCT​TCT​AGA​atg​acc​acc​cac​cgc​cta​gt3’; and reverse 5′CCC​TGA​AGT​TCT​CAG​GAT​CCt​cac​ttc​gcc​ttt​ccc​tgg​gca3′, followed by subcloning into PCGN vector with XbaI and BamHI sites. Point mutations on PGAM2 cDNA, K49R, K146R, and K176R, were generated by using the QuickChange II XL site-directed mutagenesis kit with the following oligos (only forward sequences are shown with the capital letters indicating the mutated sites): K49R, 5′cca​ccg​cta​tca​aag​atg​ccA​GGa​tag​agt​ttg​aca​tct​gct​ac3’; K146R, 5′gac​cgc​cgc​tat​gca​ggc​ttg​AGG​cct​gag​gag​ctg​cct​acc​tg3’; K176R, and 5′gga​gat​cgc​acc​taa​gat​taG​ggc​tgg​cca​gag​agt​gct​tat​tg3’.

### Cell culture and transfection

HeLa (human), C2C12 (mouse, myogenic), and P19 (mouse, cancerous) (ATCC, United States) cell lines were used in this study. HeLa cells were maintained in DMEM containing 10% FBS supplemented with 1% gentamycin. P19 cells were maintained in 1X alpha minimum essential medium (Corning, United States) containing 10% FBS supplemented with 1% gentamycin (Gibco, United States). C2C12 cells were maintained in regular growth medium (GM; DMEM containing 20% FBS and 1% penicillin and streptomycin; Gibco, United States). All C2C12 cells were used within 25 passages. All transfections were performed by using lipofectamine 2000 or 3000 according to the protocols provided by the manufacturer.

### Generation of homozygous PGAM2-K176R knock-in C2C12 and P19 cell lines

We used the CRISPR-Cas9–based homologous recombination strategy to generate homozygous PGAM2-K176R knock-in (PGAM2^K176R/K176R^) C2C12 and P19 cell lines, respectively. The following pair of complementary oligos harboring 20-nucleotide guide RNA sequences was used to generate guide RNA (gRNA): forward 5′CAC​CGATC​GCA​CCT​AAG​ATT​AAG​GC3′ and reverse 5′AAA​CGCC​TTA​ATC​TTA​GGT​GCG​ATC3’. The guide oligo was subcloned into pX458 as previously described (Zhang et al., 2020). Briefly, these two complementary oligos (100 μM) were phosphorylated by T4 polynucleotide kinase, annealed by boiling for 10 min, and left at room temperature overnight. Then, the annealed oligos were ligated into the *Bbs*I-cut pX458 by using the QuickLigase Kit. The resulting construct (pX458-K176R-KI-gRNA) was transformed into competent DH5α cells and verified by performing Sanger sequencing. The 320-bp donor DNA sequence (CAA​AAG​CGA​AAT​GTT​CCT​GTG​CCC​TTG​TCC​CCA​GGA​CCG​CCG​CTA​TGC​AGG​CTT​GAA​GCC​TGA​GG AGCT​GCC​TAC​CTG​TGA​AAG​TCT​CAA​GGA​CA C​CAT​TGCC CG​GGC​TTT​GCC​CTT​CTG​GAA​TGA​GGA​GAT​CGC​A CC​TAA​GAT​TCG​CGC​TGG​CCA​GAG​AGT​GCT​TAT​TG C​AGC​CCA​TG G​GAA​CAG​CCT​TCG​GGG​CAT​TGT​GAA​ACA​TCT​GGA​AGG​T GA​GGC​CTA​CTC​TCA​GGA​AAG​ATG​TG A​GAC​AGA​AAC​CA C​TGC​GGT​TCA​TTG​GTT​TCA​GCT​ATT​TTA ​CAG​ATG​TCA​G GG​TTT​CAG​AAG​TTG​ATG​GAG​GCA​GGA​GT) contained a K176R (underscored cgc, K→R) point mutation and a novel restriction site, HinP1, that cuts GCGC (bold) without affecting the amino acid sequence. Restriction enzyme cutting at the HinP1 cut site divided the 320-bp donor DNA sequence into two fragments of 148 and 172 bps, which were visualized as two bands between 100 and 200 bp in 1% agarose. The WT control allele was left uncut. Therefore, the HinP1 restriction site allowed us to screen for heterozygous and homozygous PGAM2^K176R/K176R^ cells. The donor DNA template was amplified by PCR using the following two oligos: forward, 5′ CAA​AAG​CGA​AAT​GTT​CCT​GTG​C3′, and reverse, 5′ACT​CCT​GCC​TCC​ATC​AAC​TTC3’. The pX458-K176R-KI-gRNA and the donor DNA template were co-transfected into C2C12 and P19 cells with lipofectamine 3000, and single green fluorescent protein (GFP)-positive clones were sorted by using flow cytometry. C2C12 and P19 cell clones homozygous for the PGAM2-K176R KI mutation were identified by using PCR and then HinP1 enzyme cutting, followed by confirmation with Sanger sequencing.

### Nickel affinity chromatography

We performed sumoylation assays *in vivo* as detailed previously (Wang et al., 2008). Then, nickel nitriloacetic acid (Ni-NTA) affinity chromatography was performed according to the protocol described previously (Kim et al., 2015b). Briefly, V5-his-tagged WT and mutant PGAM2 were transfected into HeLa and C2C12 cells in the absence or presence of the vectors encoding SUMO-1-wt, SUMO-1-ΔGG-mut, SUMO-2-wt, or SUMO-2-ΔGG-mut, as needed. After 48 h, cells were lysed and sonicated in 6 M guanidine buffer, and his_6_-tagged PGAM2 was immobilized on Ni-NTA beads and eluted by elution buffer (200 mM imidazole, 5% sodium dodecyl sulfate, 0.15 M Tris [pH 6.7], 30% glycerol, 0.72 M 2-mercaptoethanol). Western blot analysis was used for analyzing the eluted proteins with anti-V5-HRP to reveal modified proteins.

### Myogenic differentiation

Myogenic differentiation was induced in C2C12 cells by using a standard protocol. Briefly, C2C12 cells were cultured in GM containing 20% FBS for 2 days until they reached 90%–100% confluency. Then, GM was replaced by differentiation medium (DM) containing 2% horse serum to induce the myogenic differentiation of C2C12 cells. DM was replenished every 48 h, and C2C12 cells were fixed or lysed at different time points during differentiation [day 0 (no differentiation), day 2, day 4, and day 6] for immunofluorescence staining or Western blot analysis (see below).

### Immunofluorescence staining

C2C12 cells cultured on coverslips were fixed in 4% formaldehyde for 10 min, washed in 1X phosphate-buffered saline (PBS) three times, and incubated with the anti-sarcomeric α-myosin heavy chain (α-MHC) antibody MF20 (1:500) (Cat# 14-6503-82, eBioscience, United States) at 4°C overnight. After that, cells were incubated with the Alexa Fluor 547 goat anti-mouse secondary antibody. Nuclei were stained with 4′,6-diamidino-2-phenylindole (DAPI). Differentiated C2C12 cells were evaluated for the differentiation index and fusion index, respectively. The differentiation index was defined as the total number of nuclei in differentiating cells (i.e., number of α-MHC–positive cells divided by the total number of nuclei × 100%). The fusion index was defined as the number of nuclei per differentiated myotube. Cells were scored from at least three randomly selected fields per sample under ×20 magnification from at least 4 independent samples per group. Images were captured by using a confocal fluorescence microscope for image presentation and quantification.

### Western blot analysis

Western blotting was performed as previously described (Kim et al., 2015b). Briefly, 20–60 µg of whole-cell lysates were boiled and separated in 4%–12% Bis-Tris plus sodium dodecyl sulfate (SDS) polyacrylamide gel electrophoresis (PAGE) gels (ThermoFisher Scientific, United States), transferred to PVDF membranes, and incubated with antibodies against PGAM2, caveolin 3, or Myf5 at 4°C overnight. Then, membranes were incubated with the appropriate HRP-conjugated secondary antibody at room temperature for 1 h, or directly incubated with β-actin-HRP, flag-HRP, HA-HRP, or V5-HRP, as appropriate at 4°C overnight. The protein bands were visualized with chemiluminescence by using ECL Plus or Immobilon™ Western, according to the intensity of signal obtained from the preliminary tests. The specific protein bands were quantified by using software ImageJ, and protein levels were normalized to those of β-actin.

### Co-immunoprecipitation (co-IP)

Co-IP was performed as previously described (Beketaev et al., 2014; Kim et al., 2015b). Briefly, C2C12 cells were transfected with HA-PGAM2-wt and either PGAM2-wt-V5-his or PGAM2-K176R-V5-his, followed by co-IP with an anti-V5 antibody or IgG (control). The precipitates were subjected to Western blotting as described above. Immunoblotting was performed with anti-V5-HRP and anti-HA-HRP.

### RNA sequencing analysis

Total RNA was purified from cells by using Trizol reagent (ThermoFisher Scientific, United States). mRNA extraction, library preparation, quality control, sequencing, and data analysis were performed by Novogene (www.novogene.com). Briefly, mRNA was extracted from total RNA by using poly-T oligo-attached magnetic beads. A library was generated and examined with Qubit and qPCR for quantification and a bioanalyzer for quality control. Library preparations were sequenced on an Illumina platform and paired-end reads were generated. Differential expression analysis of two conditions/groups (two biological replicates per condition) was performed by using the DESeq2 R package (1.20.0). Genes with an adjusted *p*-value (padj) < 0.05 were considered differentially expressed genes (DEGs). An absolute fold-change of 2 (i.e., |log2 fold change| >1) was set as the threshold for significant differential expression.

### Reverse transcription and quantitative PCR

Total RNA was purified as mentioned above, and reverse transcription (RT) was performed using SuperScript™ First-Strand Synthesis System (Cat#18091050, ThermoFisher Scientific, United States). qPCR was performed on QuantStudio 6 Flex (Applied Biosystems). GAPDH was used as an internal control. The oligos for qPCR were designed by the primer bank (www.pga.mgh.harvard/edi/primerbank), and their sequences are shown below (all from 5′ to 3′): myh4: forward CTT​TGC​TTA​CGT​CAG​TCA​AGG​T; reverse AGC​GCC​TGT​GAG​CTT​GTA​AA; PGAM2: forward, TCT​GGA​GGC​GTT​CCT​TTG​AC; reverse, CTT​GAG​ACT​TTC​ACA​GGT​AGG​C; PGAM1: forward, AGG​ATC​GCA​GGT​ACG​CAG​A; reverse, CTC​CAG​ATG​CTT​AAC​GAT​GCC; GAPDH: forward, TGG​CCT​TCC​GTG​TTC​CTA​C; reverse, GAG​TTG​CTG​TTG​AAG​TCG​CA.

### Measurements of glycolytic and mitochondrial functions

Metabolic analysis of WT and PGAM2 mutant C2C12 cells and P19 cells was conducted by using a Seahorse XFe96 Extracellular Analyzer according to the manufacturer’s protocols. Briefly, 15,000 C2C12 cells or 10,000 P19 cells were seeded into assay plates containing normal cell culture media and were cultured in the incubator at 37°C and 5% CO_2_ overnight. The next day, the media was changed to assay media (i.e., Seahorse XF DMEM [pH 7.4] without phenol red, supplemented with 1 mM sodium pyruvate, 10 mM glucose, and 2 mM glutamine). The Agilent Seahorse Glycolytic Rate Assay Kit was used to measure the real-time extracellular acidification rate (ECAR) and oxygen consumption rate (OCR), which were used to determine the glycoPER. Three measurements of basal rates were recorded, followed by rotenone/antimycin A (Rot/AA) injections, which inhibit mitochondrial oxygen consumption and therefore the release of CO_2_-derived protons. Next, 2-depxy-d-glucose (2-DG) was injected, which inhibited glycolysis through the competitive binding of glucose hexokinase, the first enzyme in the glycolytic pathway. The Seahorse XF Glycolytic Rate Assay Report Generator and Wave software were used to calculate all parameters including PER. Additionally, the Agilent Seahorse Cell Mito Stress Test kit was used to record OCR and ECAR. OCR was recorded at baseline and at 6-min intervals after three injections (oligomycin, FCCP, and Rot/AA, respectively). Basal respiration, proton leak, maximal respiration, spare respiratory capacity, non-mitochondrial oxygen consumption, ATP production, coupling efficiency, and spare respiratory capacity (primary determinants of mitochondrial function) were calculated by using the Seahorse XF Cell Mito Stress Test Report Generator/Wave software.

### Statistical analysis

Data are presented as the mean ± standard deviation. One-way analysis of variance (ANOVA) followed by Duncan’s test was used for comparing data among multiple groups. The Mann-Whitney non-parametric *U* test was used for comparing data between two groups. A *p*-value less than 0.05 was considered statistically significant.

## Results

### PGAM2 is a SUMO-1 substrate

**FIGURE 1:**
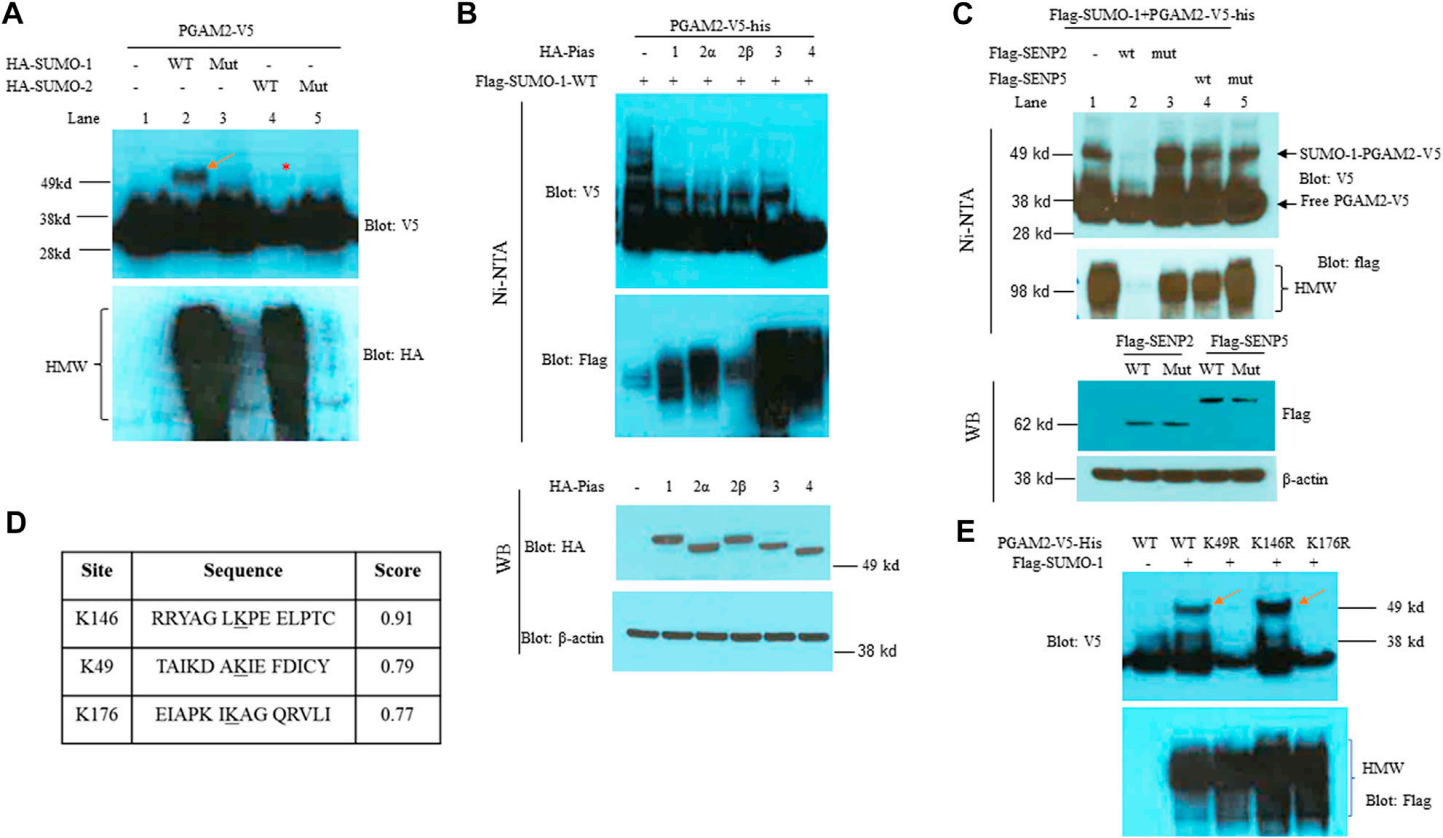
PGAM2 is a SUMO-1 substrate with SUMO acceptor sites at K49 and K146. **(A)** Results of Ni-NTA pulldown experiments performed in HeLa cells transfected with PGAM2-V5-his and one of the following expression vectors: HA-SUMO-1-wt, mut (i.e., deficient for conjugation), HA-SUMO-2-wt, or HA-SUMO-2-mut, as indicated. The top panel was probed with V5-HRP, and the bottom panel was probed with HA-HRP. Notably, the strong, slowly migrating band (red arrow) was observed only in the presence of both PGAM2-V5-his and HA-SUMO-1-wt, and the weak, slowly migrating band (red asterisk) was observed in the presence of both PGAM2-V5-his and HA-SUMO-1-wt. **(B)** Pias family proteins do not promote SUMO moiety conjugation to PGAM2. Top two panels: Ni-NTA pulldown experiments were performed in HeLa cells transfected with PGAM2-V5-his and flag-SUMO-1-wt in the presence of Pias family proteins, as indicated. Lower two panels: Western blot analysis was performed to evaluate the expression levels of HA-tagged Pias family proteins after the transfection of HeLa cells. β-actin was used as an internal control. **(C)** SENP2-wt deconjugates SUMO1-conjugated PGAM2. Ni-NTA pulldown experiments were performed as described above with the indicated expression vectors. Note that SUMO1-PGAM2 disappeared only in the lane with the presence of flag-SENP2-wt but in not others (top panel). The second panel shows that flag-SENP2-wt also abolishes global SUMO-1 conjugation. Western blot analysis was performed to reveal the comparable expression of flag-SENP2-wt, flag-SENP2-mut, flag-SENP5-wt, and flag-SENP5-mut (third panel). β-actin was used as a loading control (the bottom panel). **(D)** SUMO plot shows the different scores for the predicted sumoylation sites on PGAM2 (K146, K49, and K176). **(E)** Ni-NTA pulldown experiments were performed in HeLa cells transfected with vectors encoding PGAM2-wt or one of its point mutants, K49R, K146R, or K176R, together with flag-SUMO-1 expression vector. SUMO-conjugated PGAM2 is indicated with red arrows. HMW, high molecular weight conjugates.

We first explored whether PGAM2 is a SUMO substrate. PGAM2-V5-his was transfected into HeLa cells alone or with expression vectors encoding HA-SUMO-1-wt, HA-SUMO-1-mut, HA-SUMO-2-wt, or HA-SUMO-2-mut, respectively. When we performed Ni-NTA pulldown assays with the transfected cell line extracts, we observed a slowly migrating band in the PGAM2-V5-his + flag-SUMO-1-wt lane but not in the PGAM2-V5-his + HA-SUMO-1-ΔGG-mut lane (compare lanes 2 and 3, upper panel, red arrow, Figure 1A), indicating that PGAM2 was modified by SUMO-1. As expected, the SUMO-1-wt lane but not the SUMO-1-ΔGG-mut lane contained high molecular weight (HMW) conjugates (lower panel, compare lanes 2 and 3; Figure 1A). PGAM2 was only weakly modified by HA-SUMO-2-wt (compare lanes 2 and 4, upper panel, red asterisk, Figure 1A). These findings indicate that PGAM2 is a strong substrate of SUMO-1 but a weak substrate of SUMO-2.

SUMO conjugation may be potentiated by SUMO E3 ligases. Since Pias proteins are well recognized SUMO E3 ligases (Wang et al., 2008; Wang, 2010), and also play a role in myogenic differentiation (Diao et al., 2009), we examined whether Pias proteins function as an E3 ligase for PGAM2 sumoylation. After HeLa cells were transfected with PGAM2-V5-his + flag-SUMO-1-wt in the presence of one of five PIAS family members (Pias 1, 2α, 2β, 3, or 4), we performed Ni-NTA chromatography to examine PGAM2 sumoylation. The Pias proteins were expressed at comparable levels (bottom two panels, Figure 1B), and as expected, they enhanced global sumoylation to different extents (HMW, second panel, Figure 1B). Intriguingly, the levels of the slow-moving sumoylated band of PGAM2 were significantly decreased by Pias 1, 2α, 2β, and 3, and completely lost in the presence of Pias 4 (top panel, Figure 1B). We also examined whether SENP2 (a general peptidase) or SENP5 (a more restricted peptidase) deconjugated SUMO-1–conjugated PGAM2. Flag-SENP2-wt but not flag-SENP5-wt deconjugated SUMO-1–conjugated PGAM2 (compare lanes 2 and 4, top panel, Figure 1C). SENP2-wt also abolished global SUMO-1 conjugation (second panel, Figure 1C). Flag-SENP2-mut and flag-SENP5-mut, both enzymatically deficient and equivalently expressed, had no significant effects on PGAM2 sumoylation (lanes 3 and 5, Figure 1C).

The bioinformatics tool SUMOplot revealed three potential SUMO acceptor sites on PGAM2 at K146, K49, and K176, with a score of 0.91, 0.79, and 0.77, respectively (Figure 1D). To examine sumoylation at these sites, we mutated each lysine to arginine to generate PGAM2-K49R, PGAM2-K146R, and PGAM2-K176R mutants expressed on the V5-his vector. When we performed *in vivo* sumoylation assays, we found that K49R and K176R mutations but not the K146R mutation substantially reduced PGAM2 sumoylation (top two panels, Figure 1E). As expected, overexpression of flag-SUMO-1-wt increased global sumoylation (i.e., HMW conjugates) in each group (bottom panel, Figure 1E). These findings indicate that K49 and K176 are both primary SUMO acceptor sites on PGAM2.

### PGAM2 expression increases during myogenic differentiation

**FIGURE 2:**
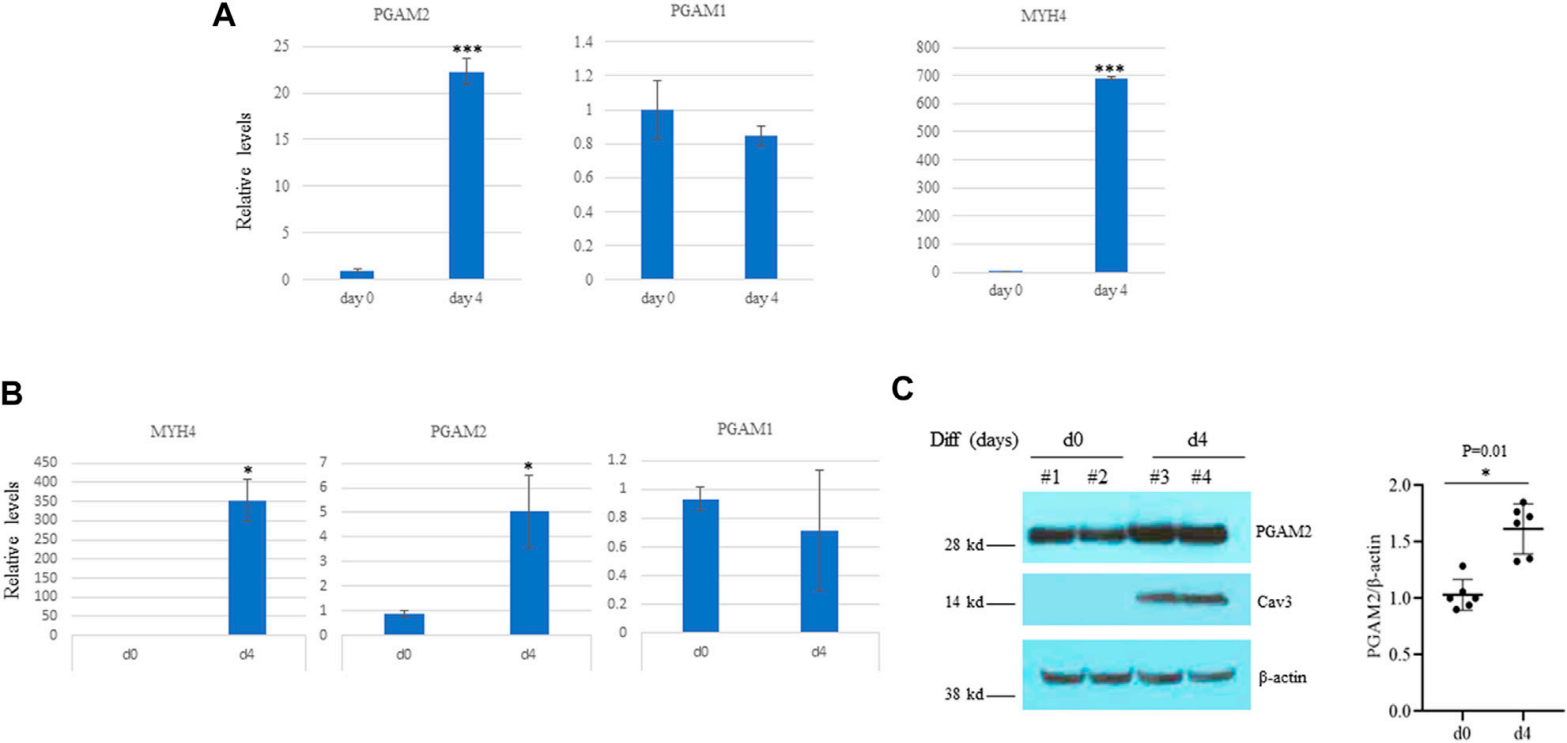
PGAM2 expression is increased during myogenic differentiation. **(A)** Results of RNA sequencing analysis showing that the expression of *PGAM2* but not *PGAM1* was significantly increased at differentiation day (d)4 when compared with d0. The increased expression of *MHC4* at d4 compared with d0 was used as a positive control. *n* = 3 per group. ****p < 0.001 *vs.* d0. **(B)** Results of real-time PCR analysis of differentiating C2C12 cells at different days confirming increased *PGAM2* expression during myogenic differentiation. *n* = 4 per group. *p < 0.05 *vs.* d0. **(C)** Protein levels of PGAM2 were significantly increased in differentiating WT C2C12 cells at day d4 compared with d0. Western blot analysis performed on whole-cell lysates purified from d0 and d4 WT C2C12 cells. Cav3 was used as a differentiation marker. β-actin was used as a loading control. *n* = 6 per group.

As mentioned above, PGAM2 is highly expressed in skeletal muscle, and mutations in PGAM2 have been linked to GSDX. However, whether PGAM2 is implicated in myogenic differentiation has remained unknown. We first examined the transcription of *PGAM2* during myogenic differentiation by performing RNA sequencing analysis. PGAM2 transcript levels were significantly increased in differentiation day (d)4 C2C12 cells compared with non-differentiated d0 cells (*p* < 0.001, Figure 2A), whereas transcript levels of *PGAM1*, another member of the PGAM family, were not significantly changed during myogenic differentiation. As expected, transcript levels of *MYH4*, a myogenic marker, were increased during myogenic differentiation (*p* < 0.001, Figure 2A). These findings were further confirmed by performing RT-qPCR) (Figure 2B). When we performed Western blot analysis to evaluate the protein levels of PGAM2 in d0 and d4 myogenic cells, PGAM2 protein levels were increased during myogenic differentiation (*p* < 0.01, Figure 2C), consistent with our findings above. Differentiation in d4 cells was marked by the appearance of the myogenic marker caveolin 3 (Cav3). Thus, our findings indicate that PGAM2 expression increases during myogenic differentiation.

### Myogenic differentiation is impaired in PGAM2^K176R/K176R^ C2C12 cells

We aligned the amino acid sequences of PGAM2 from zebrafish, human, mouse, rat, and bovine and found that K176 is conserved among all five of these species. K49 is conserved among four species (Supplementary Figure S1A). K176 but not K49 is also conserved between mouse PGAM1 and mouse PGAM2 and among human PGAM1, PGAM2, and PGAM4 (Supplementary Figures S1B, C). Because K176 is more highly conserved among paralogs and orthologs, we targeted K176 to generate a K176R knock-in mutation in myogenic C2C12 cells by using CRISPR-cas9–based homology-directed repair, to examine the physiological importance of PGAM2 sumoylation. Using the same approach, we also generated a K176R knock-in mutation in cancerous P19 cells. We obtained one homozygous K176R knock-in (PGAM2^K176R/K176R^) C2C12 cell line (clone #207), several heterozygous knock-in (PGAM2^K176R/+^) C2C12 cell lines (only clone #106 was shown), and several PGAM2^K176R/K176R^ P19 cell lines (clone #12 and #21, Supplementary Figures S2A-D).

**FIGURE 3:**
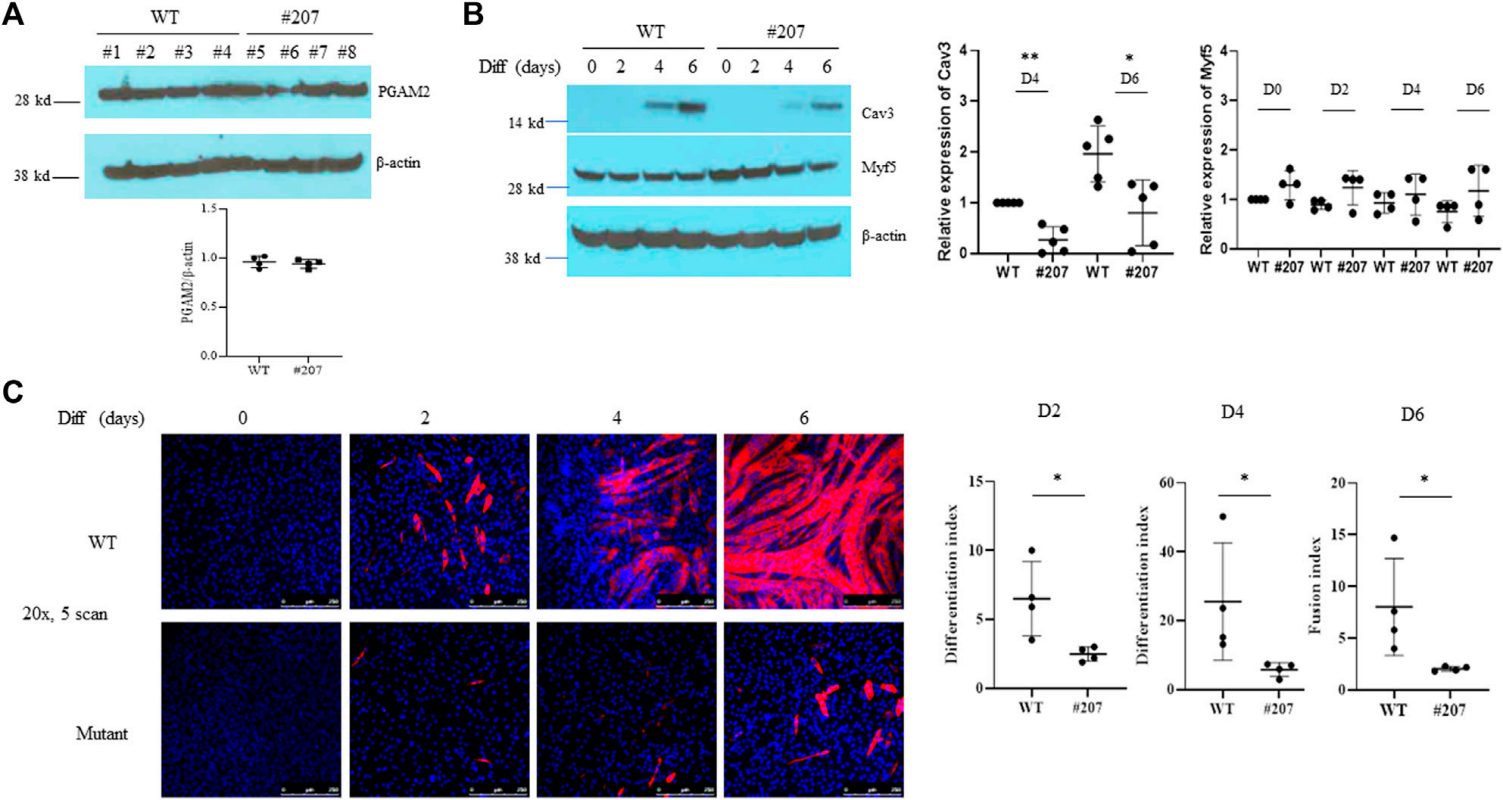
The PGAM2-K176R mutation impairs myogenic differentiation. **(A,B)** Western blot analysis of whole-cell lysates from WT and C2C12^K176R/K176R^ cells (#207) at differentiation day (d)0 (i.e., undifferentiated), d2, d4 and d6. Cells were plated and cultured in growth medium (GM) overnight in 6-cm plates and then switched to differentiation medium (DM). DM was replenished every 2 days. β-Actin was used as a control. *p < 0.05, **p < 0.01 *vs*. WT. **(C)** Immunofluorescence staining for MHC was performed in WT and C2C12^K176R/K176R^ cells at differentiation days as indicated. Red, MHC; blue, DAPI. *p < 0.05, *vs.* WT. Magnification, ×20.

We next investigated the effect of the PGAM2-K176R knock-in mutation on myogenic differentiation. Western blot analysis showed that endogenous PGAM2 levels were equivalent between WT and PGAM2^K176R/K176R^ C2C12 cells at homeostasis (Figure 3A). To evaluate the differentiation capacity of WT and PGAM2^K176R/K176R^ C2C12 cells, we compared expression levels of the myogenic marker Cav3 in protein lysates at d0, d2, d4, and d6 (Hong et al., 2016). Cav3 expression was detectable starting at d4 and increased at d6 in both WT and PGAM2^K176R/K176R^ C2C12 cells (Figure 3B); however, the expression of Cav3 was significantly lower in mutant cells than in WT cells at d4 and d6 (*p* < 0.01 and *p* < 0.05, respectively, Figure 3B). The expression of Myf5, a myogenic transcription factor critical for myogenic differentiation, was not significantly different between WT and PGAM2^K176R/K176R^ C2C12 cells (Figure 3B). These findings indicate that K176R knock-in impaired myogenic differentiation independent of Myf5.

In accordance with the above observations, immunofluorescence staining for MYH4, another widely used myogenic differentiation marker, showed that PGAM2^K176R/K176R^ C2C12 cells had a significantly lower differentiation index at d2 and d4 and a lower fusion index at d6 than did WT C2C12 cells (Figure 3C). PGAM2^K176R/+^ and WT C2C12 cells showed comparable differentiation capabilities (data not shown). Together, our findings indicate that myogenic differentiation is deficient in PGAM2^K176R/K176R^ C2C12 cells.

### The PGAM2-K176R mutation decreases the glycolytic rate of myogenic cells

**FIGURE 4:**
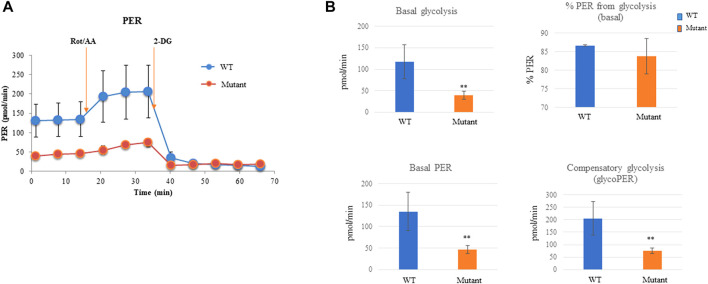
PGAM2-K176R mutation decreases the glycolytic rate. For measuring the glycolytic rate, C2C12 cells were seeded into 96-well Seahorse cell culture plates and cultured overnight. The Seahorse XFe96 Extracellular Analyzer was used to record three basal rate measurements, followed by injections with Rot/AA according to the manufacturer’s protocol. Data were automatically generated by the Seahorse XF Glycolytic Rate Assay Report Generator and Wave program. **(A)** One representative mitochondrial respiration curve from seven independent experiments is shown. **(B)** PGAM2^K176R/K176R^ mutant cells exhibited a reduced basal glycolytic rate, basal PER, and compensatory glycolysis. No significant difference was observed in % PER from glycolysis (basal) between WT and PGAM2^K176R/K176R^ mutant cells. Data are expressed as the mean ± SD. *n* = 6 wells per group. **p < 0.01 vs WT.

Given that PGAM2 plays an important role in energy production, we examined the glycolytic rates of WT and PGAM2^K176R/K176R^ C2C12 cells. PGAM2^K176R/K176R^ C2C12 cells had a significantly lower rate of basal glycolysis, basal PER, and compensatory glycolysis (glycoPER) than did the WT C2C12 cells, except that there was no difference in % PER from glycolysis (basal) (Figures 4A–C). Thus, our findings indicate that the PGAM2-K176R mutation reduces the glycolytic rate of myogenic cells. Because PGAM2 plays an important role in energy production in cancer cells (Jiang et al., 2014; Mikawa et al., 2020), we asked whether the sumoylation site K176 is also critical for PGAM2 to mediate energy production in cancer cells. We used the same strategy to generate PGAM2^K176R/K176R^ P19 cells (Supplementary Figures S2A,B), and subjected WT and PGAM2^K176R/K176R^ cells to glycolytic rate analysis. Similar findings were obtained from WT and PGAM2^K176R/K176R^ P19 cells as those from WT and PGAM2^K176R/K176R^ C2C12 cells (Supplementary Figure S3). Thus, the SUMO site K176 is also important for PGAM2 to mediate glycolysis in cancer cells.

### The PGAM2-K176R mutation impairs mitochondrial function

**FIGURE 5:**
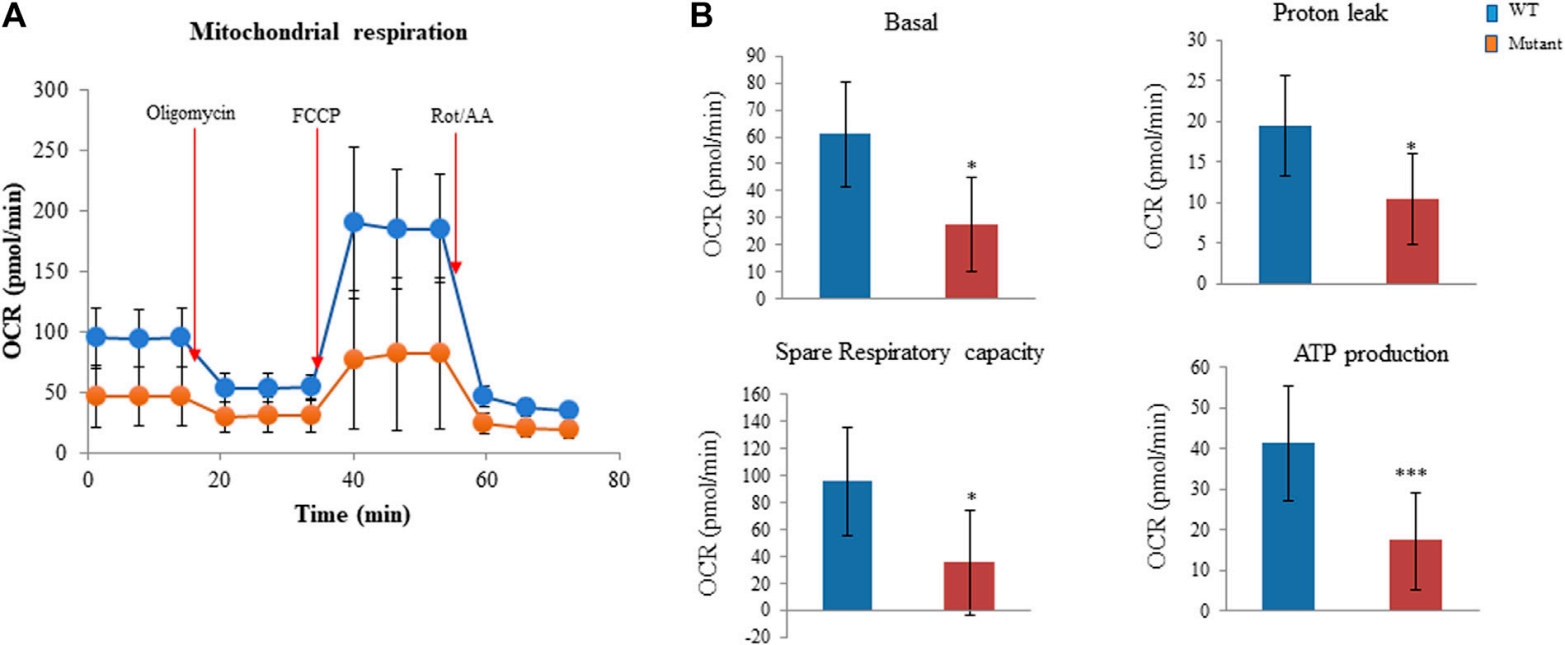
PGAM2^K176R/K176R^ mutation impairs mitochondrial function. For measuring the oxygen consumption rate (OCR), C2C12 cells were seeded into 96-well Seahorse cell culture plates and cultured overnight. The Seahorse XFe96 Extracellular Analyzer was used to record OCR at baseline and three times at 6-min intervals after injections with 5 µM oligomycin, 3 µM FCCP, and 4 µM rotenone/antimycin A (Rot/AA), respectively, according to the manufacturer’s protocol. Data were automatically generated by Seahorse XF Cell Mito Stress Test Report Generator. **(A)** One representative mitochondrial respiration curve is shown. **(B)** PGAM2^K176R/K176R^ mutant cells exhibited reduced basal OCR, spare respiratory capacity, proton leak, and ATP production compared with WT cells. Data are expressed as mean ± SD. n = 6 wells per group. *p < 0.05, ***p < 0.001 vs WT.

We also examined whether the PGAM2-K176R mutation affected mitochondrial function in C2C12 cells. Consistent with our observations that the PGAM2-K176R mutation significantly impaired cell glycolysis, we found that PGAM2^K176R/K176R^ C2C12 cells also exhibited significantly lower basal OCR, spare respiratory capacity, proton leak, and ATP production than did WT C2C12 cells (Figures 5A,B). Thus, these findings indicate that the PGAM2-K176R mutation impairs mitochondrial function.

### The homodimerization of PGAM2 is unaffected by the K176R mutation

PGAM2 functions as a homodimer (Zhang et al., 2001; Mikawa et al., 2014). Because the K176R mutation impaired the function of PGAM2, we determined whether this mutation impaired the homodimerization of PGAM2. Co-IP experiments were performed on lysates from C2C12 cells transfected with HA-PGAM2-wt and either PGAM2-wt-V5-his or PGAM2-K176R-V5-his by using V5 antibody–conjugated agarose beads. We found that comparable amounts of HA-PGAM2-WT were precipitated by V5-tagged PGAM2-wt and PGAM2-K176R (Supplementary Figure S4), indicating that the K176R mutation does not affect PGAM2 homodimerization.

### Gene expression is dysregulated in PGAM2^K176RK176R^ C2C12 cells at d0

**FIGURE 6:**
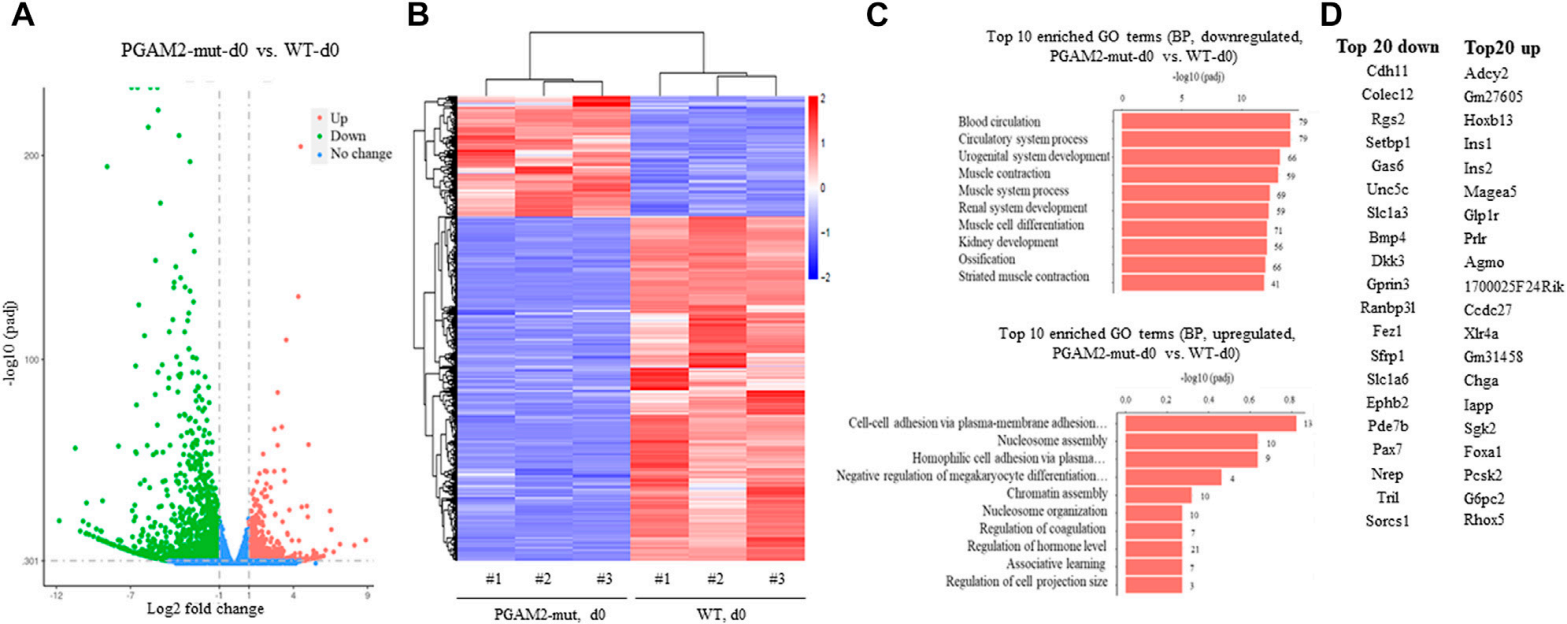
Dysregulated gene expression in PGAM2-K176R mutant C2C12 cells at differentiation day 0. Results of RNA sequencing analysis of WT and PGAM2-K176R mutant C2C12 cells at differentiation day (d)0. **(A)** Volcano plot with a filter of padj <0.05 and a |log2 fold change| = 1 shows the changes in differentially expressed genes (DEGs) in WT and PGAM2-K176R mutant C2C12 cells. **(B)** Heatmap providing a global view of DEGs in WT and PGAM2-K176R mutant cells. **(C)** Top 10 most enriched GO terms in the biological pathways (BPs) associated with downregulated (top panel) and upregulated (bottom panel) genes in PGAM2-K176R mutant cells compared with WT cells. **(D)** Top 20 most downregulated (left panel) and upregulated (right panel) genes in PGAM2-K176R mutant cells compared with WT cells.

To systemically examine the transcription profile of PGAM2^K176RK176R^ C2C12 cells, we performed RNA sequencing analysis of WT and PGAM2^K176R/K176R^ C2C12 cells at d0 and d4. We used a filter of padj <0.05 and a |log2 fold change| = 1. At d0, PGAM2^K176RK176R^ C2C12 cells had 452 upregulated and 1289 downregulated genes compared with WT C2C12 cells (Figure 6A). In Figures 6A,B heatmap provides a global view of differentially expressed genes (DEGs) in WT and mutant cells at d0. When we subjected these DEGs to pathway analysis, we found that the top 20 biological pathways (BPs) associated with the genes downregulated in the mutant cells included muscle/skeletal/striated muscle development and muscle cell migration/differentiation (top panel, Figure 6C). On the other hand, the major BPs affected by genes upregulated in mutant cells were ribonucleoprotein complex/ribosome biogenesis and mitochondrion organization (bottom panel, Figure 6C). The top 20 most upregulated and downregulated genes in PGAM2^K176R/K176R^ C2C12 cells are listed in Figure 6D. Consistent with these cells exhibiting deficient myogenic differentiation, some important genes that drive myogenic differentiation, such as *Unc5c*, *Ranbp31*, and *Pax7*, were downregulated at d0 in PGAM2^K176RK176R^ C2C12 cells. On the other hand, some myogenic differentiation suppressor genes such as *HOXB13* were upregulated at d0.

### Gene expression is dysregulated in PGAM2^K176RK176R^ C2C12 cells at d4

**FIGURE 7:**
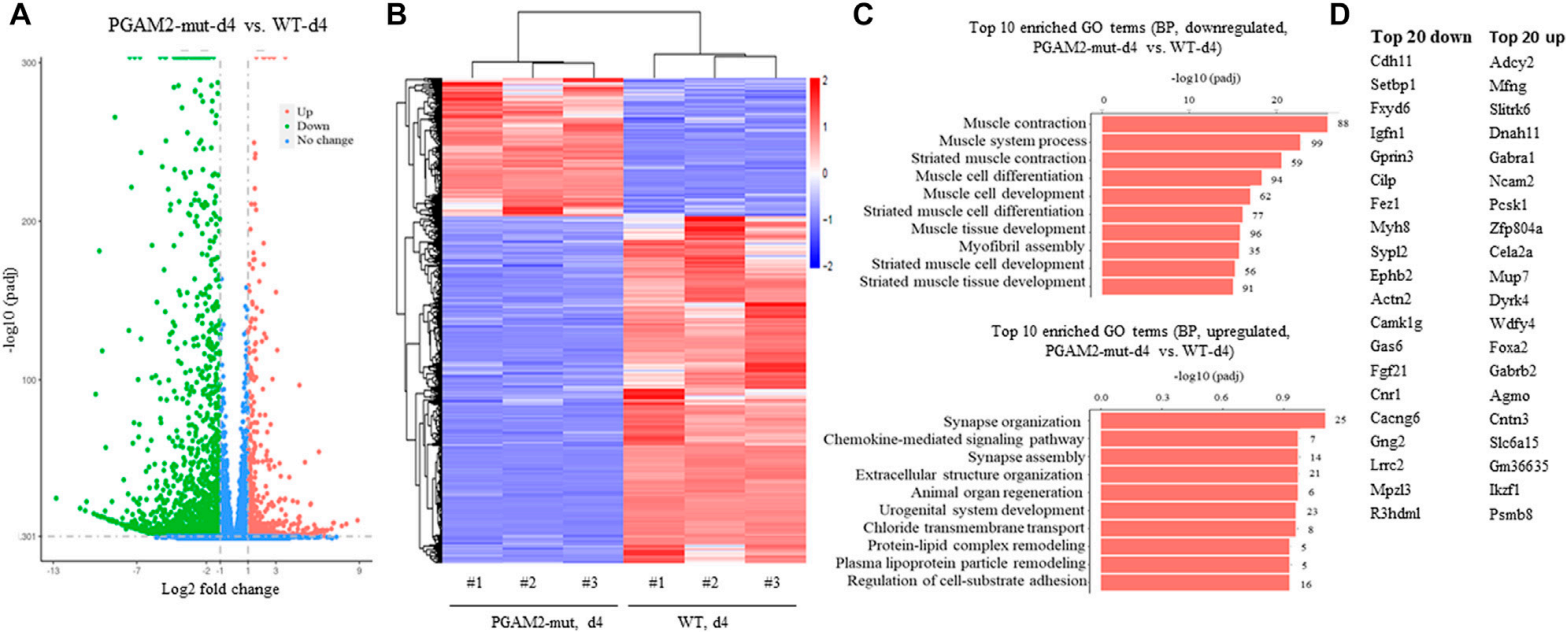
Dysregulated gene expression in PGAM2-K176R mutant C2C12 cells at differentiation day 4. Results of RNA sequencing analysis for WT and PGAM2-K176R mutant C2C12 cells at differentiation day (d)0. **(A)** Volcano plot with a filter of padj <0.05 and a |log2 fold change| = 1 shows the changes in differentially expressed genes (DEGs) in WT and PGAM2-K176R mutant C2C12 cells. **(B)** Heatmap providing a global view of DEGs in WT and PGAM2-K176R mutant cells. **(C)** Top 10 most enriched GO terms in BPs associated with downregulated (top panel) and upregulated (bottom panel) genes in PGAM2-K176R mutant cells compared with WT cells. **(D)** Top 20 most downregulated (left panel) and upregulated (right panel) genes in PGAM2-K176R mutant cells compared with WT cells.

We examined the transcription profile of WT and PGAM2^K176RK176R^ C2C12 cells at d4 and found that PGAM2^K176RK176R^ C2C12 cells had 700 upregulated and 1747 downregulated genes compared with WT C2C12 cells (Figure 7A). In Figures 7A,B heatmap provides a global view of DEGs in WT and PGAM2-K176R C2C12 cells. When we subjected these DEGs to pathway analysis, the top 20 BPs associated with the downregulated genes in PGAM2^K176R/K176R^ cells included muscle/skeletal/striated muscle development and muscle cell migration/differentiation (top panel, Figure 7C). On the other hand, the major BPs affected by the upregulated genes in PGAM2^K176RK176R^ cells were ribonucleoprotein complex/ribosome biogenesis and mitochondrion organization (bottom panel, Figure 7D). The top 20 most upregulated and downregulated genes in PGAM2^K176RK176R^ C2C12 cells are listed in Figure 7D. These data suggest that, compared with WT C2C12 cells, PGAM2-K176R C2C12 cells exhibit deficient muscle-related programs at d4.

### Naturally occurring missense mutations of PGAM2 impair sumoylation

**FIGURE 8:**
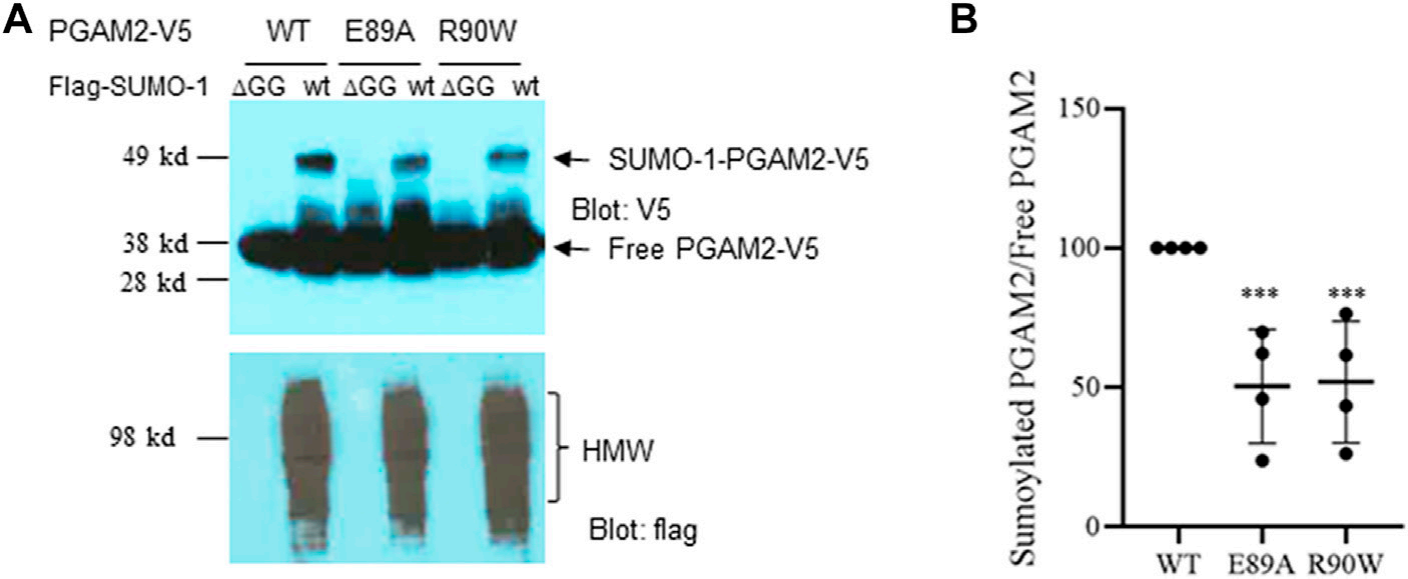
PGAM2 with missense mutations linked with human disease exhibit reduced sumoylation. A Ni-NTA pulldown experiments were performed on C2C12 cells transfected with PGAM2-V5-wt, PGAM2-V5-E89A, or PGAM2-V5-R90W in the presence of flag-SUMO-1-wt or flag-SUMO-1-ΔGG mutant, respectively. Top panel, anti-V5 blot; bottom panel, anti-flag blot. HMW, high molecular weight conjugates. **(B)** Statistical analysis of data shown in **(A)**. Data for sumoylated PGAM2 were normalized to those for free PGAM2, and PGAM2-V5-wt sumoylation was given a value of 100%. *n* = 4 per group. ***p < 0.001 vs WT.

As mentioned above, PGAM2 mutations have been linked to GSDX in humans. Most of those mutations are caused by premature termination of translation; two of them, E89A and R90W, are missense mutations (Naini et al., 2009). We examined whether these two mutations affect PGAM2 sumoylation. Indeed, Ni-NTA chromatography performed with extracts from C2C12 cells transfected with PGAM2-V5-wt, PGAM2-V5-E89A, or PGAM2-V5-R90W together with flag-SUMO-1-wt or flag-SUMO-1-mut revealed that either of these two mutations (E89A or R90W) reduced PGAM2 sumoylation by approximately 50% (*p* < 0.01 *vs.* WT, Figures 8A,B).

## Discussion

PGAM2 has been well studied for its role in human GSDX, and its activity is known to be mediated primarily by PTMs. In this study, we identified sumoylation, a versatile type of PTM, as a novel PTM of PGAM2 at two major SUMO acceptor sites, K49 and K176. The mutation of PGAM2 K176 to R176 impaired the differentiation capacity of myogenic cells, which was accompanied by compromised glycolysis and mitochondrial function. These findings indicate that the sumoylation of PGAM2 is important for myogenic cells to maintain their full capacity for terminal differentiation and myoblast fusion, as well as for normal energy production.

## PGAM2 and SUMO

We showed that PGAM2 is a novel SUMO substrate at two primary SUMO acceptor sites, K49 and K176, indicating another regulatory layer for the fine-tuning of PGAM2 activity. Notably, the mutation of one lysine abolished sumoylation of the other. Conceivably, the mutation of one site may alter the structure of PGAM2, thereby reducing the exposure of the other SUMO site to the SUMO machinery. Alternatively, sumoylation of one acceptor site may be required for sumoylation of the other site. In addition, we found that PGAM2 was primarily a target of SUMO-1 but only a weak target of SUMO-2, supporting the notion that SUMO-1 and SUMO-2 have differential targets and may potentially play different roles in cellular events.

### PGAM2 sumoylation and myogenic differentiation

PGAM2 is a muscle-specific enzyme; however, its role in myogenic differentiation was not previously explored. To examine how sumoylation affects the role of PGAM2 in myogenic differentiation, we generated a PGAM2-K176R knock-in C2C12 cell line. PGAM2^K176RK176R^ C2C12 cells were able to terminally differentiate, but at a significantly reduced capacity, as indicated by lower levels of Cav3 expression and lower differentiation and fusion indexes than in WT C2C12 cells. At the same time, the expression of Myf5, an important factor for myogenic differentiation, was comparable between WT and PGAM2^K176RK176R^ C2C12 cells. Therefore, we speculated that PGAM2 sumoylation, at least on K176, is required for full myogenic differentiation and myotube fusion, but that PGAM2 mediates downstream effectors or signaling independently of Myf5 to modulate myogenic differentiation. Given our findings, w further hypothesize that PGAM2 is required for myogenic differentiation, which can be studied in the future by generating PGAM2-null C2C12 cells and mouse.

In this study, we mutated only K176 and not K49 because we found that K176 is more highly conserved across orthologs and paralogs than is K49, although this does not rule out the possibility that K49 is also important for terminal myogenic differentiation. Because of technical difficulties, we were not able to detect the sumoylation of endogenous PGAM2 during myogenic differentiation. However, in a recent study in which mass spectrometry was used to identify SUMO targets during adipocyte differentiation, five potential SUMO sites were discovered on PGAM2, including K176 (Zhao et al., 2022). This suggests that the sumoylation of K176 is involved in adipocyte differentiation, which is consistent with our findings. Previous studies in which the role of SUMO substrates in myogenic activity was examined involved the overexpression of sumoylation-deficient substrates such as Pax7, MyoD, and G9a (Luan et al., 2013; Joung et al., 2018; Srinivasan et al., 2019). This study is the first to our knowledge in which a single SUMO site knock-in mutation of an endogenous substrate has been analyzed in C2C12 cells to study the effect of a sumoylation-deficient substrate on myogenic differentiation in a physiologic setting.

As noted, myogenic differentiation was accompanied by increased PGAM2 expression. However, the expression of PGAM1, the brain isoform of PGAM, did not significantly increase during myogenic differentiation. These observations suggest that PGAM1 and PGAM2 may play different roles in myogenic differentiation. Whether and how the differential expression of PGAM1 and PGAM2 contributes to their respective roles in myogenic differentiation or even myogenesis *in vivo* remains unknown and merits further exploration in the future.

### PGAM2 sumoylation and energy production

Myogenic differentiation requires high mitochondrial activity (Wust et al., 2018), accompanied by increased glycolysis (Kim et al., 2010; Fortini et al., 2016). Moreover, glycolysis promotes myoblast fusion during embryogenesis *in vivo* (Tixier et al., 2013), and the inhibition of mitochondrial activity with chloramphenicol in QM7 cells (an avian myoblast cell line) or with ethanol in monkey myoblasts impaired myogenic differentiation (Rochard et al., 2000; Levitt et al., 2020). In addition, increased Warburg-like glycolysis was shown to be associated with activated satellite cells, which are myogenic stem cells, after muscle injury (Fu et al., 2015). These findings collectively suggest the positive role of metabolic activation in myogenic differentiation. Although we found that the K176R mutation of PGAM2 reduced the capability of myogenic cells to terminally differentiate, the exact underlying mechanisms was unclear. Given the important role of mitochondrial function and glycolysis in myogenic differentiation and PGAM2’s role in glycolysis, we examined the effects of PGAM2-K176R mutant on the glycolytic rate and mitochondrial stress with the widely adopted Seahorse glycolytic rate and mitochondrial stress assays. We found that PGAM2^K176R/K176R^ C2C12 cells exhibited a lower glycolytic rate than did WT C2C12 cells, as demonstrated by their reduced basal glycolysis, reduced basal PER, and reduced compensatory glycolysis. No significant difference was seen in % PER from basal glycolysis, indicating that glycolysis remained the primary energy production program in PGAM2^K176R/K176R^ cells. In addition, compared with WT C2C12 cells, PGAM2^K176R/K176R^ C2C12 cells exhibited compromised mitochondrial function, as revealed by decreased basal OCR, spare respiratory capacity, proton leak, and ATP production. Thus, we postulate that deficient mitochondrial function and impaired glycolysis underlie the compromised terminal differentiation of PGAM2^K176R/K176R^ C2C12 myogenic cells.

In the present study, we also found that the PGAM2-K176R mutation impaired glycolysis in P19 cells, a cancer cell line. PGAM2 exhibits a well-defined role in the Warburg effect (Mikawa et al., 2020), which is closely associated with cancer growth and metastatic progression. It is of great interest to investigate further whether PGAM2^K176R/K176R^ P19 cells have impaired cell growth and/or compromised metastatic capability.

## Changes in transcription profiles and BPs in PGAM2-K176R mutant cells

We systemically analyzed the transcription profiles of WT and PGAM2^K176R/K176R^ C2C12 cells at two different time points during differentiation: d0 (undifferentiated) and d4. Given the differentiation deficiency of PGAM2^K176R/K176R^ C2C12 cells, we predicted that at d0, the genes that drive myogenic differentiation would be downregulated in these cells. Indeed, *Unc5c*, *Ranbp31*, and *Pax7* were among the top 20 most downregulated genes in d0 mutant cells. Unc5c is a receptor of netrin-1, which promotes myogenic differentiation and myotube formation in satellite cells, which are skeletal muscle stem cells (Suzuki et al., 2021). Ranbp31 is a nuclear export factor that inhibits bone morphogenetic protein (BMP) signaling and thus promotes myogenic differentiation (Friedrichs et al., 2011; Chen et al., 2015). Depletion of Pax7, a transcription factor that governs myogenesis, impairs the myogenic differentiation of satellite cells (Seale et al., 2000; von Maltzahn et al., 2013). In addition, the increased expression of HOXB13, a homeobox transcription factor, suppresses myogenic differentiation (Aspuria et al., 2020). Consistent with the results of our immunofluorescence staining and Western blot analysis showing the deficient differentiation of PGAM2-K176R mutant cells at d4, we found that four of the top 20 BPs that were associated with downregulated genes were muscle related, with involvement in muscle contraction, muscle system process, muscle cell differentiation, and striated muscle contraction. These observations were further supported by our evidence that *Myh8*, another myogenic marker (Tosic et al., 2018), was among the top 20 most downregulated genes at d4 in PGAM2^K176R/K176R^ C2C12 cells. Together, these findings support the premise that the SUMO site K176 of PGAM2 is essential for the full terminal differentiation of myogenic cells.

### PGAM2 sumoylation deficiency and genetic disease

Several naturally occurring mutations of genes that are involved in human genetic diseases have been reported to alter sumoylation. For example, congenital heart disease–linked *Nkx2.5* mutations impaired sumoylation (Wang et al., 2008), and *Nkx2.5* haplo-insufficient mice with the cardiac-specific expression of sumoylation-deficient *Nkx2.5* mutant developed congenital heart defects (Kim et al., 2011), recapitulating the human cardiac phenotypes. Similar findings were obtained in studies of *Zic3*, a gene important for normal left–right patterning in humans (Chen et al., 2013). In our study, we found that two naturally occurring human missense mutations of PGAM2 resulting in E89A and R90W amino acid replacements significantly reduced PGAM2 sumoylation. These mutations of PGAM2, like the others identified in *Nkx2.5* and *Zic3*, did not affect direct SUMO moiety acceptors; actually, some were quite distant from the primary sumoylation site. A plausible explanation is that these mutations caused conformational changes in the proteins, thereby altering the sumoylation of substrates.

## Data Availability

The data presented in the study are deposited in the NCBI/GEO repository, accession number GSE220249.
