# Supplementary material for: Sumoylation-deficient phosphoglycerate mutase 2 impairs myogenic differentiation
Source: Front Cell Dev Biol. 2022 Dec 14;10:1052363. doi: 10.3389/fcell.2022.1052363 (PMC9795042; doi:10.3389/fcell.2022.1052363)
Supplement: Supplementary file 1 [file DataSheet2.pdf]

Alignment of Pgam2 from different species

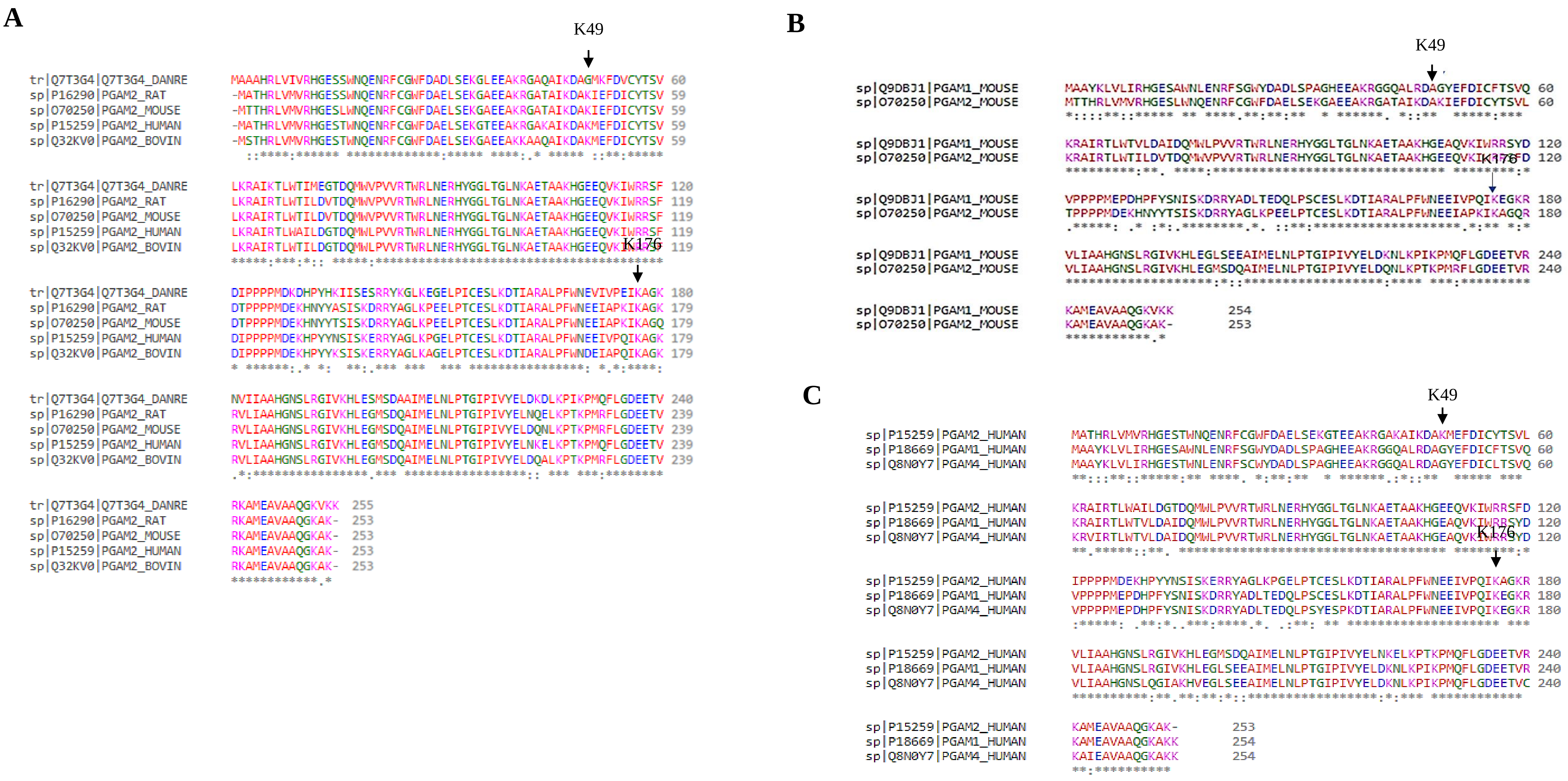

Supplemental Fig. 1

Generation of K176R KI cell line

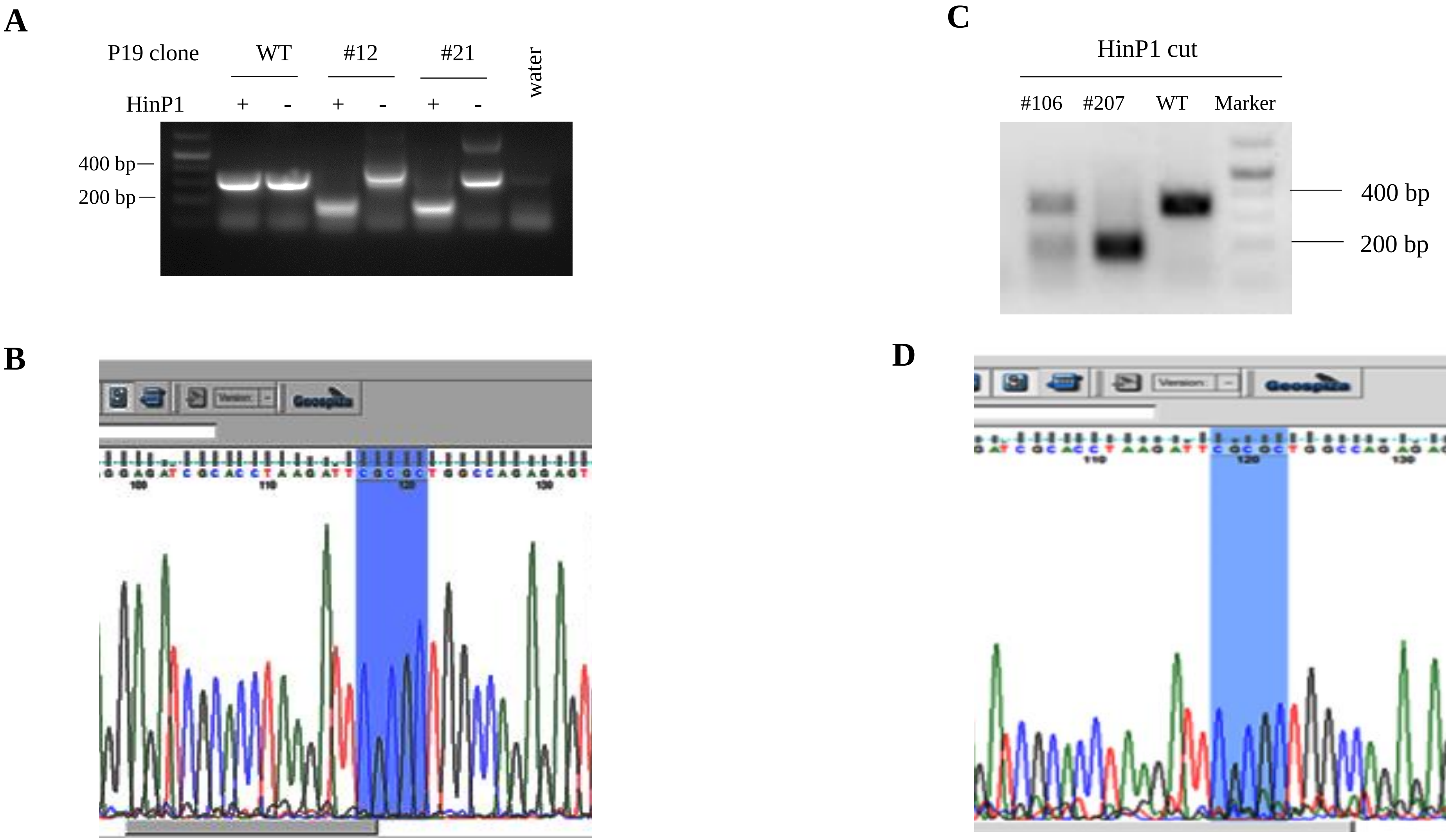

Supp Fig. 2

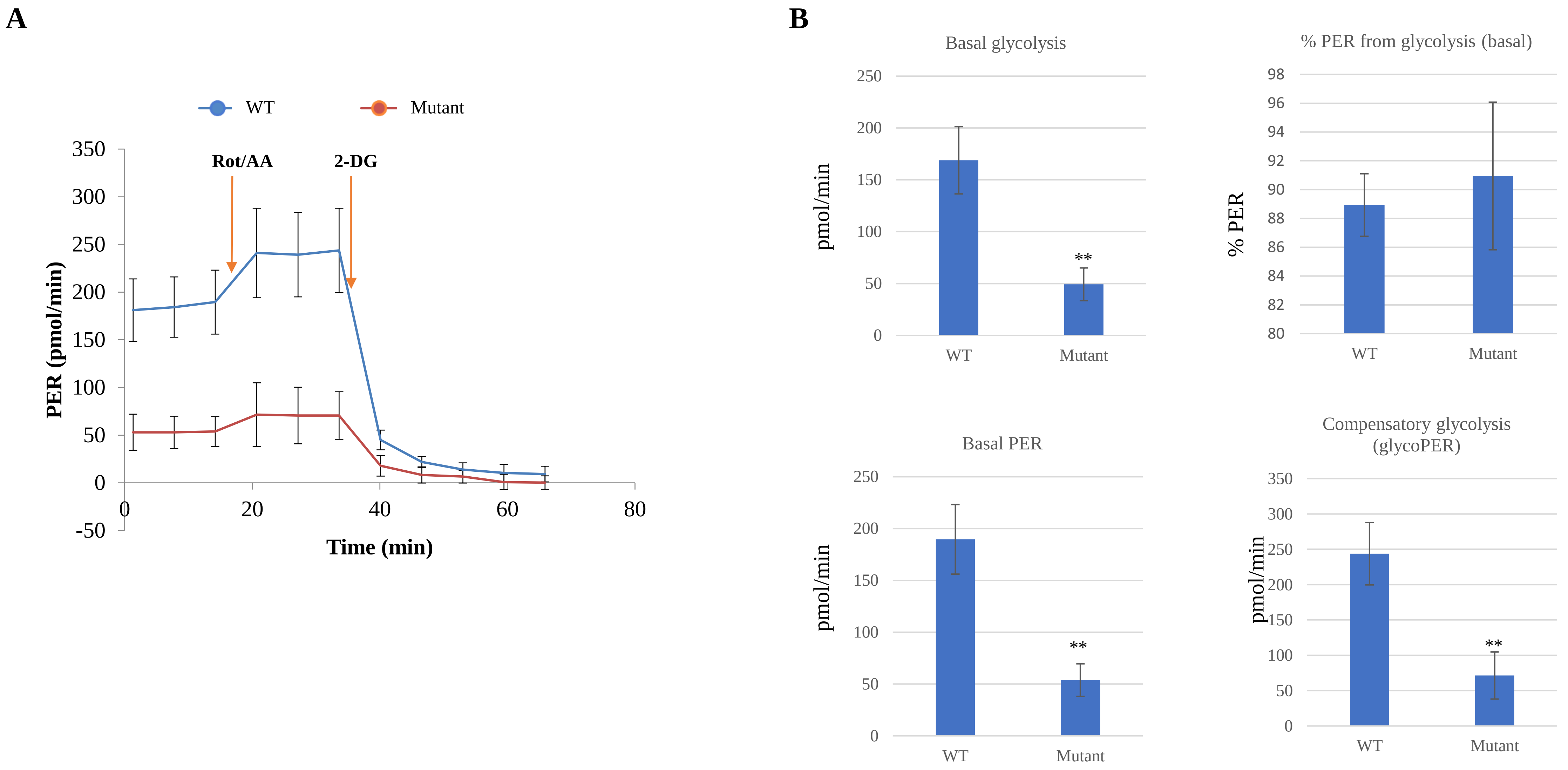
A
WT
Mutant
Rot/AA
2-DG
PER (pmol/min)
350
300
250
200
150
100
50
0
-50
0
20
40
60
80
Time (min)
B
Basal glycolysis
pmol/min
250
200
150
100
50
0
WT
Mutant
**
% PER from glycolysis (basal)
% PER
98
96
94
92
90
88
86
84
82
80
WT
Mutant
Basal PER
pmol/min
250
200
150
100
50
0
**
WT
Mutant
Compensatory glycolysis
(glycoPER)
pmol/min
350
300
250
200
150
100
50
0
**
WT
Mutant

Supp Fig. 3

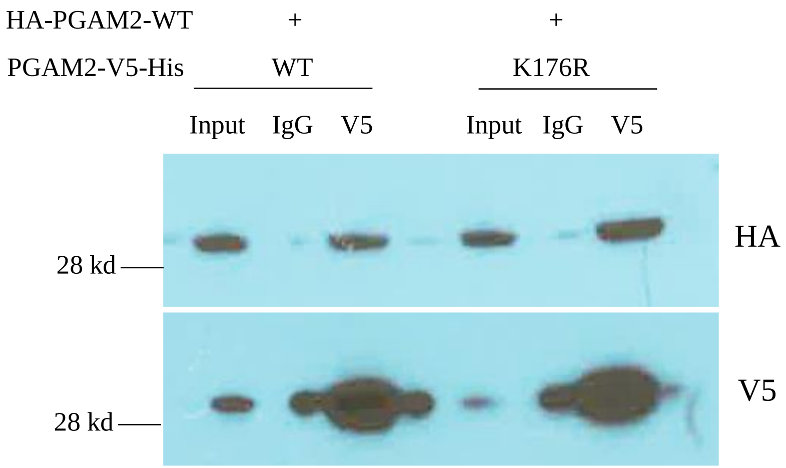
HA-PGAM2-WT
+
+
PGAM2-V5-His
WT
K176R
Input
IgG
V5
Input
IgG
V5
28 kd
HA
28 kd
V5

Supp Fig. 4
