# Supplementary material for: Sumoylation-deficient phosphoglycerate mutase 2 impairs myogenic differentiation
Source: Front Cell Dev Biol. 2022 Dec 14;10:1052363. doi: 10.3389/fcell.2022.1052363 (PMC9795042; doi:10.3389/fcell.2022.1052363)

**SUPPLEMENTARY INFORMATION**

**Supplemental Fig. 1 Evolutionary conservation of SUMO sites K49 and K176 in PGAM2 paralogs and orthologs. A** Alignment of PGAM2 ortholog sequences in Danre (Zebrafish), rat, mouse, human and bovine. **B** Alignment of mouse PGAM1 and PGAM2 sequences. **C** Alignment of human PGAM1, PGAM2, and PGAM4 sequences. Clustal Omega ([www.ebi.ac.uk](http://www.ebi.ac.uk)) was used for sequence alignment.

**Supplemental Fig. 2 Generation of K176R knock-in (KI) cell lines. A** Genotyping of two homozygous K176R knock-in P19 cell lines, #12 and #21. PCR was performed on genomic DNA purified from WT cells, clone #12, and clone #21, followed by incubation with or without HinP1 for two hours. Water was used as a negative control. **B** Sanger sequencing performed on PCR product confirmed the presence of the K176R mutation. Only clone #12 is shown. **C** Genotyping of K176R KI C2C12 cell lines #106 (heterozygous KI) and clone #207 (homozygous KI) with HinP1. For genotyping, PCR was performed on genomic DNA purified from WT cells, clone #106, and clone #207, followed by incubation with HinP1 for two hours. Of note, clone #106 has two DNA bands after HinP1 cutting, whereas clone #207 has one short band after HinP1 cutting. **D** Sanger sequencing performed on PCR product from clone #207 confirmed the presence of the K176R mutation.

**Supplemental Fig. 3 Impaired glycolysis in PGAM2^K176R/K176R^ mutant P19 cells.** WT and PGAM2^K176R/K176R^ P19 cells were seeded into 96-well Seahorse cell culture plates and cultured overnight before glycolytic measurements were recorded with the Seahorse XFe96 Analyzer. Three measurements of basal rates were recorded, followed by Rot/AA injections according to the manufacturer’s protocol. Data were automatically generated by the Seahorse XF Glycolytic Rate Assay Report Generator. **A** One representative mitochondrial respiration curve from three independent experiments is shown. **B** PGAM2^K176R/K176R^ mutant cells exhibited reduced basal glycolysis, basal PER, and compensatory glycolysis. No significant difference in % PER from glycolysis (basal) was observed between WT and PGAM2^K176R/K176R^ mutant cells. Data are expressed as mean ± SD. n=6 wells per group. **p<0.01 vs. WT.

**Supplemental Fig. 4 Homodimerization of PGAM2 unaffected by K176R mutation.** Co-immunoprecipitation was performed with anti-flag antibody on whole-cell lysates collected from C2C12 cells overexpressing flag-PGAM2 and HA-PGAM2, followed by Western blot analysis with anti-HA antibody.

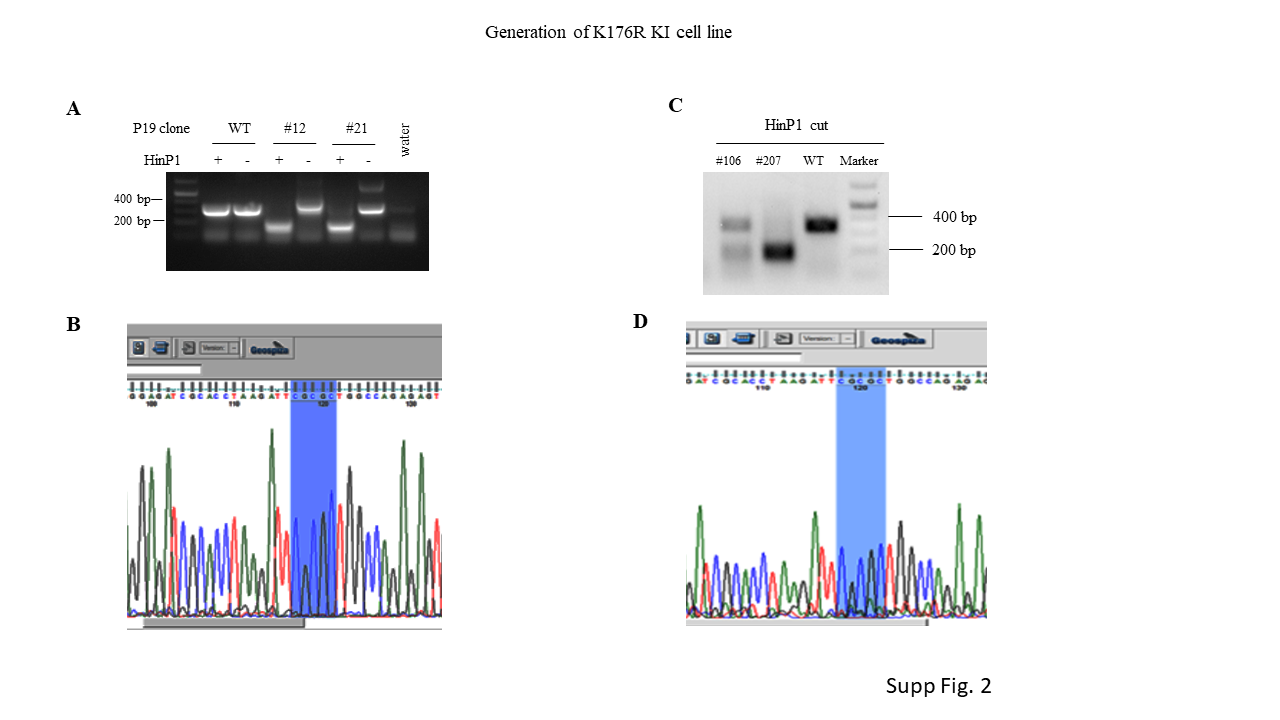


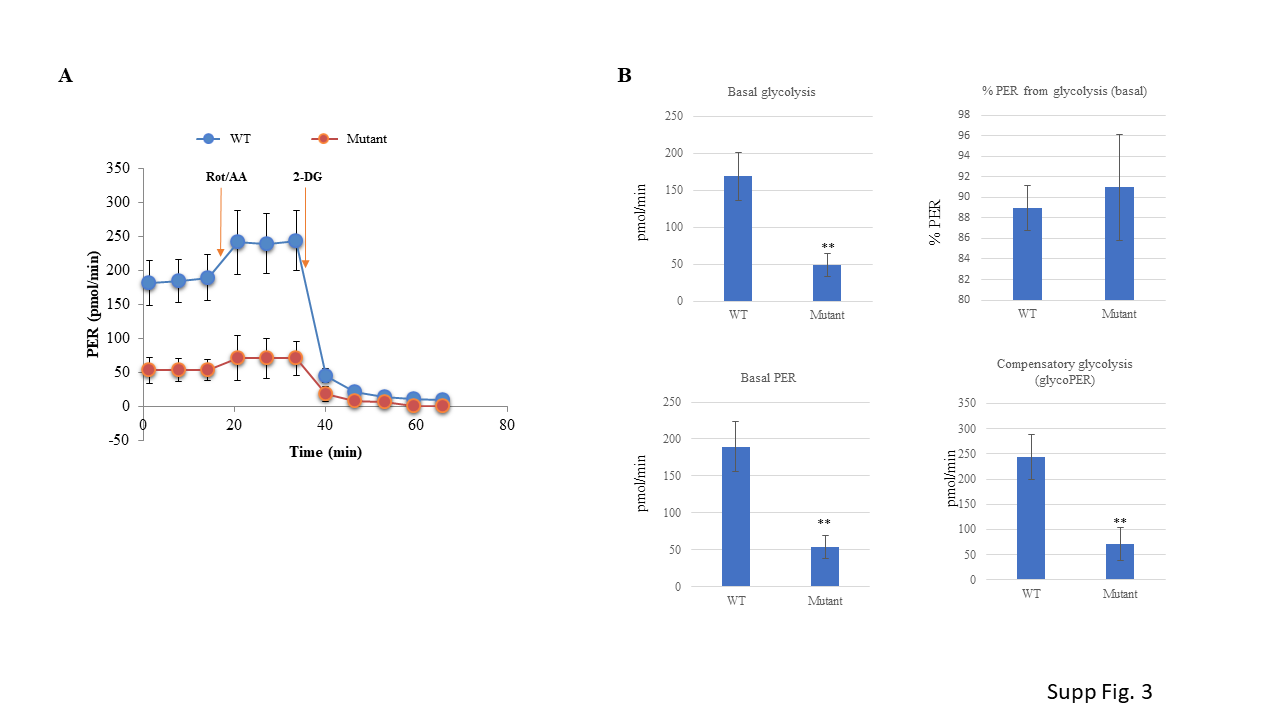


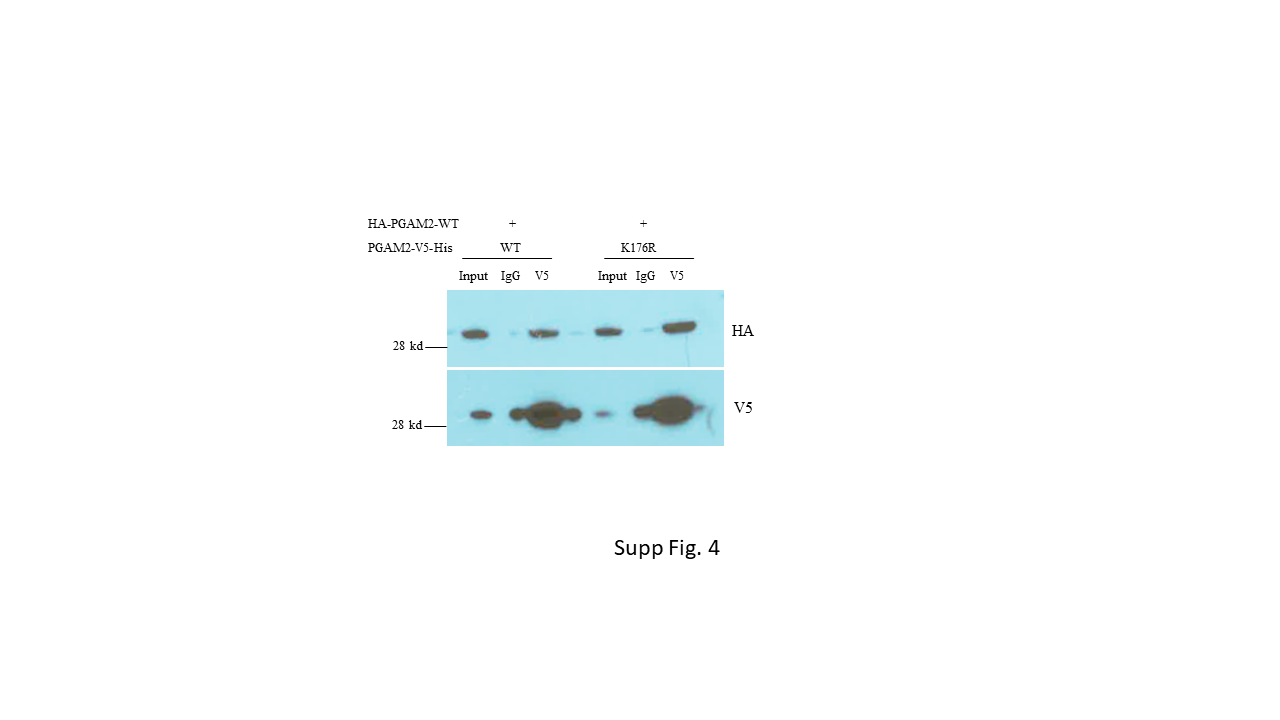

Supplement: Supplementary file 2 [file DataSheet1.docx]
